# Poly(ethylene glycol)*-block-*poly(sodium 4-styrenesulfonate) Copolymers as Efficient Zika Virus Inhibitors: *In Vitro* Studies

**DOI:** 10.1021/acsomega.2c07610

**Published:** 2023-02-09

**Authors:** Paweł Botwina, Magdalena Obłoza, Piotr Bonarek, Krzysztof Szczubiałka, Krzysztof Pyrć, Maria Nowakowska

**Affiliations:** † Virogenetics Laboratory of Virology, Malopolska Centre of Biotechnology, Jagiellonian University, Gronostajowa 7a, 30-387 Krakow, Poland; ‡ Microbiology Department, Faculty of Biochemistry, Biophysics and Biotechnology, Jagiellonian University, Gronostajowa 7, 30-387 Krakow, Poland; § Department of Physical Chemistry, Faculty of Chemistry, Jagiellonian University, Gronostajowa 2, 30-387 Krakow, Poland; ∥ Department of Physical Biochemistry, Faculty of Biochemistry, Biophysics and Biotechnology, Jagiellonian University, Gronostajowa 7, 30-387 Krakow, Poland

## Abstract

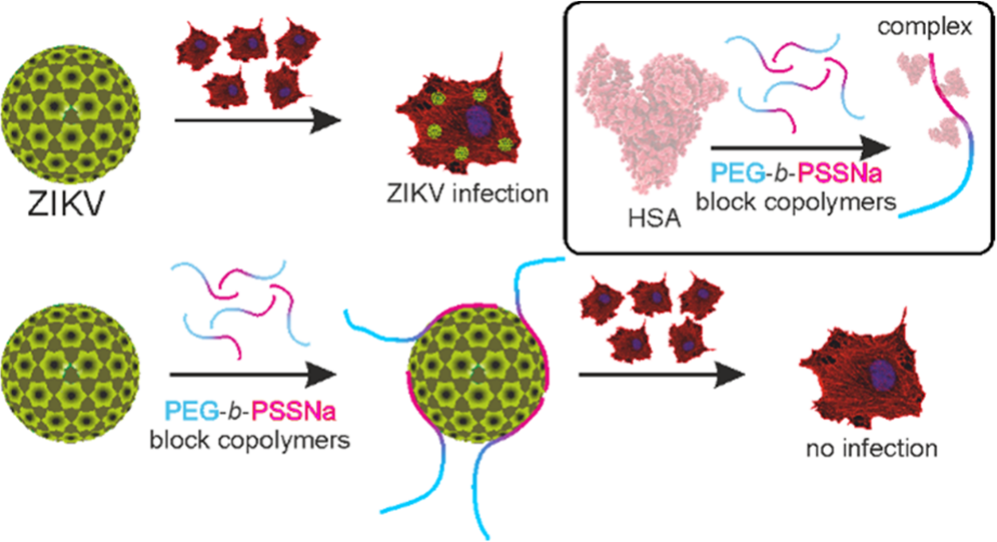

A series of poly(ethylene glycol)-*block*-poly(sodium 4-styrenesulfonate) (PEG-*b*-PSSNa) copolymers were synthesized, and their antiviral activity against Zika virus (ZIKV) was determined. The polymers inhibit ZIKV replication *in vitro* in mammalian cells at nontoxic concentrations. The mechanistic analysis revealed that the PEG-*b-*PSSNa copolymers interact directly with viral particles in a zipper-like mechanism, hindering their interaction with the permissive cell. The antiviral activity of the copolymers is well-correlated with the length of the PSSNa block, indicating that the copolymers’ ionic blocks are biologically active. The blocks of PEG present in copolymers studied do not hinder that interaction. Considering the practical application of PEG-*b*-PSSNa and the electrostatic nature of the inhibition, the interaction between the copolymers and human serum albumin (HSA) was evaluated. The formation of PEG-*b*-PSSNa-HSA complexes in the form of negatively charged nanoparticles well-dispersed in buffer solution was observed. That observation is promising, given the possible practical application of the copolymers.

## 1 Introduction

The Zika virus (ZIKV) is a single-stranded, positive-sense RNA virus belonging to the flavivirus genus along with the West Nile virus, dengue virus, yellow fever virus, and tick-borne encephalitis virus.^1^ ZIKV is an arbovirus, and the most common transmission route employs *Aedes* mosquitoes, prevalent in warm tropical and subtropical regions.^2^ ZIKV was first isolated from a rhesus monkey in 1947 and one year later from an *Aedes* sp. mosquito in the Zika Forest in Uganda.^3^ Only 14 human cases were identified during the next five decades. However, in 2007, the State of Yap (Micronesia) unexpectedly experienced an outbreak of cases, with 73% of the population demonstrating symptoms of illness.^4^ The next outbreak, involving 32,000 people, occurred in Polynesia in 2013.^5^ Ever since, ZIKV has swiftly spread over the globe. The largest outbreak began in May 2015 in Brazil and affected up to 1,300,000 people.^6,7^ On February 1, 2016, WHO classified ZIKV as a Public Health Emergency of International Concern (PHEIC) due to its rapid spread and potential sequelae.

A proportion of ZIKV-infected individuals remain asymptomatic; however, at the same time, some cases may be severe and life-threatening.^8^ Importantly, high-risk groups may be defined. When the infection occurs in a pregnant woman, the virus passes the placental barrier and may infect the fetus, causing congenital defects such as microcephaly.^6,9,10^ Infection with ZIKV is also linked to an increased risk of Guillain–Barré syndrome, encephalitis, or myelitis in adults.^11^ Until now, there is no vaccine or specific treatment for ZIKV infection. Furthermore, no compounds have reached the stage of clinical trials.

Polymers are employed in therapy as polymer–protein conjugates, drug–polymer conjugates, gene delivery systems, drug delivery systems, among other applications. Notably, polymers, both synthetic and natural, are effective antiviral inhibitors as shown in numerous studies. Human immunodeficiency virus (HIV), herpes simplex virus (HSV), influenza virus (IAV), respiratory syncytial virus (RSV), dengue virus (DENV), yellow fever virus (YFV), coronaviruses (including SARS-CoV-2), hepatitis B virus (HBV), and human papillomavirus (HPV) were all found to be hampered by polymeric compounds.^12−18^ The presence of many identical molecular motifs combined in one large polymer molecule enables simultaneous multipoint binding interaction between the polymer and the viral surface or between the polymer and the viral receptor on the cell surface. Based on a similar operating principle to a zipper, this mechanism gives antiviral polymer systems a distinct advantage over low-molecular-weight drugs.^19^

Our previous study showed that poly(sodium 4-styrenesulfonate) (PSSNa) effectively inhibits ZIKV *in vitro*. The antiviral activity strongly depends on the length (and, consequently, on molecular weight) of the PSSNa chain. PSSNa acts mainly by directly binding to ZIKV particles, thus preventing their attachment to the host cells.^20^ However, in treating ZIKV infections, PSSNa would have to be delivered intravenously and preferably cross the blood–brain barrier. To improve the pharmacokinetic properties of PSSNa, a poly(ethylene glycol) (PEG) block is attached to the PSSNa chain. PEGylation of high-molecular-weight drugs is commonly utilized to improve *in vitro* and *in vivo* pharmacokinetic parameters, i.e., enhance circulation time, increase stability, improve aqueous solubility, inhibit aggregation, and lower immunogenicity.^21,22^ In this work, a series of pegylated PSSNa block copolymers (PEG-*b*-PSSNa) was synthesized, and their antiviral properties toward ZIKV were examined and compared with those of PSSNa polymers. Our results have shown that PSSNa contained in PEG-*b*-PSSNa retains its antiviral activity, low cytotoxicity, and mechanism of action. The interactions of PEG-*b*-PSSNa with human serum albumin (HSA), a model protein, result in the formation of well-defined, nanometric-sized, negatively charged molecular complexes, which can form stable dispersion in aqueous media.

## 2 Materials and Methods

### 2.1 Reagents

Poly(ethylene glycol) 4-cyano-4-(phenylcarbonothioylthio)pentanoate (PEG-CTA, average *M*_n_ = 2000 and 10,000 Da) and 4,4′-azobis(4-cyanovaleric acid) (ACVA) were purchased from Sigma-Aldrich and used as received. Sodium 4-vinylbenzenesulfonate (sodium styrenesulfonate, SSNa) was purchased from AK Scientific and used without further purification. Methanol was purchased from POCh. Water was purified with a Millipore Milli-Q System.

### 2.2 Synthesis of PEG*_n_-b*-PSSNa*_m_*

#### 2.2.1 Preparation of PEG-*b*-PSSNa Polymers

**Figure 1:**
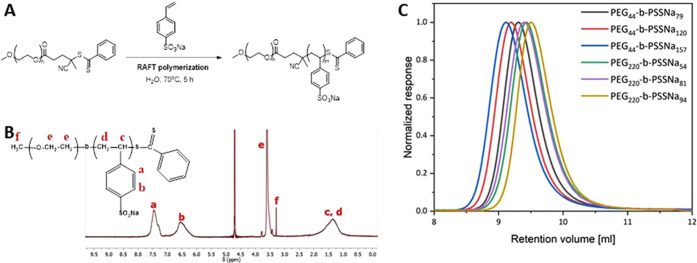
(A) Reaction scheme. (B) ^1^H NMR spectrum of PEG_220_-*b*-PSSNa_81_ copolymer in D_2_O. (C) GPC chromatograms (RALS response) of PEG-*b*-PSSNa copolymers collected at a 0.8 mL/min flow rate. A 0.1 M NaNO_3_ aqueous solution containing 20% v/v acetonitrile was used as an eluent.

PEG-*b*-PSSNa were prepared *via* a reversible addition–fragmentation chain-transfer (RAFT) polymerization of 4-vinylbenzenesulfonate (SS). The concentrations of SS, PEG-CTA, and ACVA are summarized in Table 1. A representative example of copolymer synthesis is as follows: predetermined amounts of SSNa (1 g, 4.85 mmol), PEG-CTA (97 mg, 48.5 μmol), and ACVA (2.7 mg, 9.7 μmol) were placed in a Schlenk flask in 5 mL of water. The solution was degassed by purging with argon for 30 min. Polymerization was carried out at 70 °C for 5 h and then quenched by air. The conjugate was purified by dialysis in 3.5 kDa dialysis tubing against MilliQ water and recovered by a freeze–drying technique. The reaction progress was followed by Fourier transform infrared (FTIR) spectroscopy measurements (Figure S1). The products were characterized by ^1^H NMR and gel permeation chromatography (GPC) measurements (Figure 1). The M*_n_* and *M*_w_/*M_n_* values determined by GPC and ^1^H NMR measurements are presented in Table 2. The polymers were >95% pure, as confirmed by GPC analysis (see Figure 1C).

**Table 1:** Polymerization Conditions for PEG*_n_*-*b*-PSSNa*_m_*

	concentration		
samples	[SSNa] (mM)	[PEG-CTA] (μM)	[ACVA] (μM)
PEG_44_-*b*-PSSNa_79_	1	10.0	2.0
PEG_44_-*b*-PSSNa_120_	1	6.5	1.3
PEG_44_-*b*-PSSNa_157_	1	5.0	1.0
PEG_220_-*b*-PSSNa_54_	1	10.0	2.0
PEG_220_-*b*-PSSNa_81_	1	6.5	1.3
PEG_220_-*b*-PSSNa_94_	1	5.0	1.0

**Table 2:** Number- and Weight-Average Molecular Weight (*M_n_* and *M*_w_), Molecular Weight Distribution DI (*M*_w_/*M_n_*), and Degree of Polymerization (DP) of the PSSNa Block in PEG-*b*-PSSNa Copolymers

polymer	*M_n_* (NMR)^a^ (Da)	*M_n_* (Da)	*M*_w_ (Da)	DI	DP (NMR)
PEG_44_-*b*-PSSNa_79_	18,300	10,300	11,700	1.14	79
PEG_44_-*b*-PSSNa_120_	27,000	15,700	17,800	1.13	120
PEG_44_-*b*-PSSNa_157_	34,000	19,700	22,700	1.15	157
PEG_220_-*b*-PSSNa_54_	22,000	9,700	11,600	1.19	54
PEG_220_-*b*-PSSNa_81_	27,000	11,800	14,200	1.20	81
PEG_220_-*b*-PSSNa_94_	30,000	16,700	20,200	1.21	94

### 2.3 Apparatus

^1^H NMR spectra were recorded on a Bruker Advance III 600 MHz spectrometer in deuterated solvents. FTIR spectra were recorded using a ThermoScientific Nicolet iS10 spectrometer. Gel permeation chromatography (GPC) analysis was performed using a Malvern OMNISEC CHR7100 chromatograph at room temperature with a right angle light scattering (RALS) detector and a flow rate of 0.8 mL/min. A 0.1 M NaNO_3_ aqueous solution containing 20% v/v acetonitrile was used as an eluent. The molecular weights of the polymers were calculated based on poly(4-styrenesulfonate) standard calibration. A Malvern Zetasizer Nano ZS instrument working at 173° detection angle was used in dynamic light scattering (DLS) measurements that were performed at 25 °C. General purpose mode was used as a size distribution analysis algorithm, and the reported data represent the averages from three series of measurements (10–100 runs each) and their standard deviations (mean ± SD, *n* = 3).

### 2.4 Isothermal Titration Calorimetry

Isothermal titration calorimetry (ITC) measurements were performed using a VP-ITC instrument (MicroCal, Northampton, MA). All experiments were conducted in duplicate in PBS at 37 °C. All solutions were degassed for 5 min under a vacuum before the experiments. Typically, 5 μL aliquots of 200 μM PSSNa or PEG-*b*-PSSNa solution were added as 25–30 injections into a calorimeter cell of 1435.5 μL volume filled with 20 μM HSA solution. The injection rate was 0.5 μL/s, and the interval between injections was 3 min. To ensure proper mixing after each injection, a constant stirring speed of 300 rpm was maintained throughout the experiment. Data analysis was performed using MicroCal Origin scientific plotting software.

### 2.5 Cells and Virus

U251 (Human Glioblastoma), Vero (*Cercopithecus aethiops* kidney epithelial, ATCC CCL-81), and primary human skin fibroblast (HSF) cells were cultured in Dulbecco’s modified Eagle’s medium (DMEM; high glucose, Corning) supplemented with 10% heat-inactivated fetal bovine serum (FBS; Life Technologies).

H/PF/2013, MR776, PRAVABC59, Human/2015/Honduras, and Mosquito/1966/Malaysia Zika virus strains were obtained from BEI Resources. For ZIKV stocks, subconfluent Vero cells were infected at TCID_50_ = 400/mL and maintained for 3 days. The cells were lysed by three freeze–thaw cycles, and the virus-containing medium was collected, aliquoted, and kept at 80 °C. The stock was titrated and the TCID_50_ value was assessed according to the Reed and Muench formula. Parallelly, a mock sample was prepared in an identical manner from noninfected cells.

### 2.6 XTT Assay

The XTT Cell Viability Assay kit (Biological Industries Cromwell, CT) was performed as described before.^20^ Briefly, the cells were incubated with PEG-*b*-PSSNa for three days at 37 °C. The medium was removed after incubation, and 100 μL of fresh medium was applied to the cells. Then, the activated XTT reagent (25 μL) was added, and the cells were incubated for 2 h. The absorbance (λ = 450 nm) was measured with a SpectraMAX 250 spectrophotometer (Molecular Devices, San Jose, CA). The data were reported as a signal ratio (in percent) of the tested sample and the control sample (solvent-treated cells).

### 2.7 Antiviral Assay

Appropriately diluted copolymers (50 μL, 2× final concentration) were applied to the cells, which were then immediately infected with 2000 TCID50/ml ZIKV (50 μL, 2 times final concentration). At 2 h postinfection, the cells were washed thrice with PBS and then incubated with polymers in fresh media for 3 days at 37 °C. Supernatants were collected, and the number of ZIKV RNA copies was assessed using RT-qPCR.

A series of mechanistic tests were carried out to establish at which stage the ZIKV replication process is hampered by PSSNa or PEG-*b*-PSSNa.

(a) The “Virus inactivation assay” verified the ability of the compound to inactivate the virus. PSSNa or PEG-*b*-PSSNa (250 μg/mL) was incubated with virions (TCID50 = 1,000,000/mL) for 10, 15, 30, and 60 min at room temperature with mixing. Then, the samples were diluted 1000 times to dilute compounds below their active concentration. The samples were titrated on confluent Vero cells according to the Reed–Muench formula, as described before.^23^
(b) The “Cell Protection Assay” verified whether the polymer interacts with the host cell and protects it from the infection. For 30 min at 37 °C, the cells were treated with 100 μL of PSSNa or PEG-*b*-PSSNa (250 μg/mL) in growth media. The cells were then rinsed three times with PBS before being infected with ZIKV (TCID50 = 10,000/mL). The development of CPE was then observed under a light microscope after 24, 48, and 72 h.
(c) The “Virus Attachment Assay” evaluates whether the polymer prevents the attachment of virus particles to the host cell. The cells were precooled to 4 °C before being inoculated with 100 μL of PSSNa or PEG-*b*-PSSNa (250 μg/mL) and 100 μL of ZIKV (TCID50 = 10,000/mL). To enable virus attachment while preventing its internalization, the cells were incubated at 4 °C. After that, the cells were rinsed three times with ice-cold PBS and 100 μL of fresh medium was added. The development of CPE was then observed under a light microscope after 24, 48, and 72 h.
(d) The “Virus Replication, Assembly, and Egress Assay” was used to determine if the compound inhibits the ZIKV replication at later stages of the infection. To allow the virus to enter the cells, 100 μL of ZIKV (TCID50 = 10,000/mL) was inoculated onto cells and incubated for 2 h at 37 °C. After incubation, the cells were rinsed three times with PBS and 100 μL of PSSNa or PEG-*b*-PSSNa polymers was added to the growth medium. The development of CPE was then observed under a light microscope after 24, 48, and 72 h.

### 2.8 RNA Isolation and RT-qPCR

A commercially available RNA isolation kit (Viral DNA/RNA Isolation Kit, A&A Biotechnology, Poland) was used to isolate viral RNA according to the manufacturer’s protocols using KingFisher Flex Purification System (Thermo Fisher). The GoTaq Probe 1-Step RT-qPCR System Protocol kit was used for reverse transcription (RT) and quantitative real-time PCR (RT-qPCR) isolated RNA (Promega, Madison). Because highly charged polymers have been shown to impact the RNA isolation process, the supernatants were diluted 1000-fold before isolation.^28^ The RT-qPCR reaction was carried out with 3 μL of isolated viral RNA, which was reverse transcribed and amplified in a 10 μL reaction containing 1× GoScript TM RT Mix for 1-Step RT-qPCR, 1× GoTaq Probe qPCR Master Mix with dUTP, 300 nM specific probe labeled with 6-carboxyfluorescein (FAM), and 6-carboxytetramethylrhodamine (5′ FAM-CGG CAT ACA GCA TCA GGT GCA TAG GAG-TAMRA-3′) and 450 nM of each primer (5′ TTG GTC ATG ATA CTG ATT GC 3′ and 5′ CCT TCC ACA AAG TCC CTA TTG C 3′). The following settings were used to run the reaction in a thermal cycler (Bio-CFX96 Rad’s Touch Real-197 Time PCR Detection System): 15 min at 45 °C (reverse transcription), 2 min at 95 °C, and then 40 cycles of 15 s at 95 °C and 30 s at 60 °C. To determine the number of viral RNA molecules in the sample, appropriate standards were prepared.

## 3 Results

### 3.1 Polymers

Applying the reversible addition–fragmentation chain-transfer (RAFT) polymerization, a series of well-defined PEG-*b*-PSSNa copolymers with narrow molecular weight dispersity (*D* ≤ 1.21) was synthesized (see Figures 1A and S1 and Table 2). The copolymers were characterized by the number-average molecular weights in the range of 18,000–34,000 Da. Two subgroups differing in the length of PEG blocks were prepared. The PEG blocks in three of the copolymers obtained were composed of 44 EG mers, while in three others they were considerably longer and contained 220 EG units. The macromolecules in each of these subgroups differed in the length of the PSSNa blocks.

The polymer compositions were confirmed by ^1^H NMR and attenuated total reflectance-FTIR (ATR-FTIR) (Figures 1B and S2 and S3). The M*_n_* and *M*_w_/*M_n_* values were determined by GPC and ^1^H NMR measurements (Figure 1B,C and Table 2). The degree of polymerization (DP) was calculated based on ^1^H NMR spectra by comparing integrals of **c** and **d** proton signals (Figure 1B).

The self-assembly behavior of PEG-*b*-PSSNa copolymers in aqueous solutions at various pH was studied. Data for PEG_44_-*b*-PSSNa_157_ and PEG_220_-*b*-PSSNa_94_ and for PSSNa_141_ are presented in Table S1. The self-assembly of these macromolecules is mainly controlled by a balance between electrostatic and hydrophobic interactions. The macromolecules of block copolymers form nanoparticles of comparable sizes at given pH.

### 3.2 Cytotoxicity

**Figure 2:**
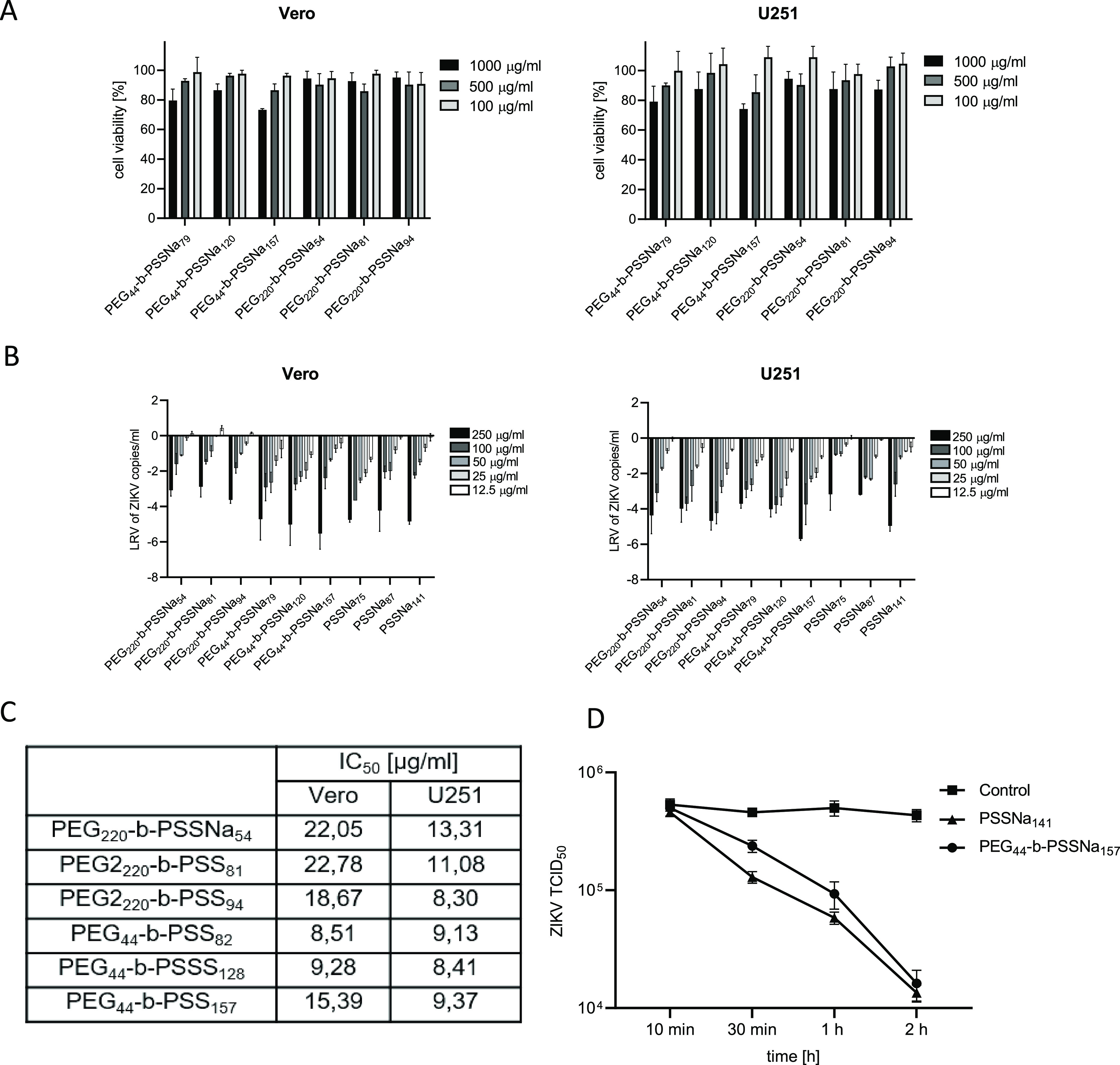
PEG-*b*-PSSNa and PSSNa are not cytotoxic and hamper ZIKV replication *in vitro*. (A) Cytotoxicity of PEG-*b*-PSSNa of various molecular weights at 1000, 500, and 100 μg/mL. Results of XTT assay of the tested polymers on Vero and U251 cells. All experiments were performed in triplicate. Average values with standard deviations (error bars) are presented. (B) Inhibition of ZIKV H/PF/2013 replication cycle by PEG-*b*-PSSNa and PSSNa. The assay was carried out in the Vero and U251 cells infected with the ZIKV H/PF/2013 virus in the presence of different polymers at given concentrations. Inhibition of the infection was evaluated using RT-qPCR. Data are shown as the average logarithmic reduction values (LRV) of ZIKV RNA copy number per milliliter with SEM (error bars). All experiments were performed in triplicate. (C) Experimental values of half-maximal inhibitory concentration (IC50) of PEG-*b*-PSSNa in Vero and U251 cells. (D) Virus inactivation assay results after ZIKV incubation with polymers (100 μg/mL).

The first experiment was to compare whether the addition of a PEG block would affect the cytotoxicity of PSSNa. For this purpose, the XTT colorimetric test was performed. Cytotoxicity of PEG-*b*-PSSNa copolymers of various molecular weights was tested using a wide range of concentrations: 1000, 500, and 100 μg/mL using Vero and U251 cells. It was observed that up to 500 μg/mL, the copolymers were not toxic. A nonsignificant decrease in viability was observed for the copolymers with the longest PSSNa block at 1000 μg/mL (a decrease of approximately 30% in PEG_44_-*b*-PSSNa_157_) (Figure 2A). This is consistent with previous observations for PSSNa polymers.^20^ Moreover, the introduction of a longer PEG_220_ block did not affect the overall cytotoxicity (Figure 2A).

### 3.3 Anti-ZIKV Properties

The antiviral properties of PEG-*b*-PSSNa toward ZIKV were tested and compared to those of the PSSNa homopolymer of similar molecular weight. The results showed that PEGylation of PSSNa did not impair their anti-ZIKV properties. The copolymers retained their dose-dependent inhibition, drastically reducing the ZIKV viral copy number in the Vero and U251 supernatants by more than 4 LRV at the highest concentrations tested (250 μg/mL) (Figure 2B). IC_50_ doses are similar for all tested polymers and ranged between 8 and 13 μg/mL in U251 and between 8 and 23 μg/mL in Vero cells (Figure 2C). As in the case of the PSSNa homopolymer, we observed a correlation between the ZIKV proliferation inhibition and the PSSNa chain length. Importantly, we have observed that the PEG chain length does not influence these properties. The comparison of the efficiency of ZIKV inactivation by PSSNa homopolymer and PEG-*b*-PSSNa copolymer with a similar number of SSNa units in the chain indicated that ionic interactions play a crucial role in that process (Figure 2D).

We have previously shown that the PSSNa polymer interacts directly with the ZIKV virion and sterically blocks virus adsorption to the host cell. The same mechanism has been shown for feline herpesvirus type 1.^24^ Due to the structural properties of the anionic block of PEG-*b*-PSSNa and PSSNa, the postulated PEG-*b*-PSSNa mechanism should not differ from that previously described for PSSNa. Cell assays examining at which stage PEG-*b*-PSSNa inhibits the virus’s replication cycle confirmed this hypothesis. PEG-*b*-PSSNa lowered the infectivity of ZIKV after incubation with the copolymer. The inhibition efficiency increased over time and was the most pronounced after 2 h incubation. No differences were observed in the CPE development between the cells treated with the tested copolymers and the cells infected without the addition of the compounds.

**Figure 3:**
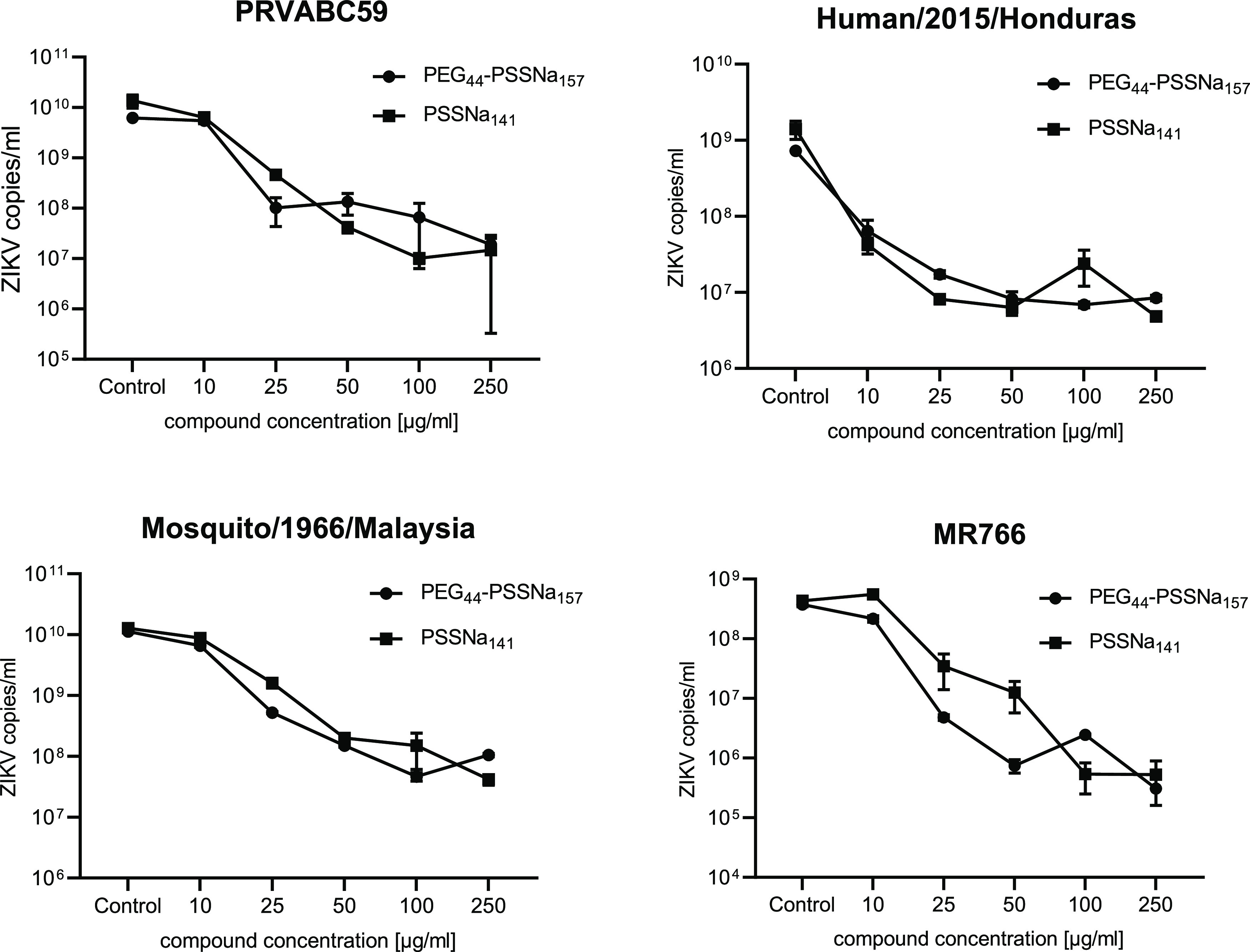
Inhibition of the ZIKV replication by PEG-*b*-PSSNa and PSSNa. U251 cells were infected with different virus strains in the presence or absence of different polymers and incubated for four days. Inhibition of the infection was evaluated using RT-qPCR. The results are presented as average values of three replications with SEM (error bar).

To confirm the hypothesis that PEGylation did not worsen the anti-ZIKV properties of PSSNa, the performance of the polymers was also tested using different strains. PEG-*b*-PSSNa inhibited the multiplication of various ZIKV strains, reducing the number of viral RNA copies by up to 3 LRV in U251 cells (Figure 3). PEG_44_-*b*-PSSNa_157_ and PSSNa_141_ show similar activity at the same weight concentration. However, taking into account that the molecular weight of PEG_44_-*b*-PSSNa_157_ is by about 24% higher than that of PSSNa_141_, it is PEG_44_-*b*-PSSNa_157_ that seems to be more active than PSSNa_141_ in terms of molar concentration.

### 3.4 Interaction of PEG-*b*-PSSNa with Human Serum Albumin (HSA)

The interaction of PEG-*b*-PSSNa with HSA was studied using a dynamic light scattering technique (DLS) and isothermal titration calorimetry (ITC). The results of these studies (see Tables 3 and 4) indicated that macromolecules of the polymers studied interact with HSA forming well-defined molecular PEG-*b*-PSSNa + HSA aggregates. In the buffer solution, they exist as negatively charged nanoparticles with dimensions in the range of 5–15 nm (Table 3). The highly negative values of their ζ-potential suggest that they can form stable dispersion in an aqueous environment (Table S2). Interestingly, the pegylation of PSSNa increases the value of ζ-potential of the polymer-HSA aggregates, thus making the system more stable. That can be explained considering the effect of PEG on the conformation of the macromolecules affecting the charge distribution (similar results were obtained for another model protein–bovine serum albumin (BSA, Table S3)).

**Table 3:** Dimension, Dispersity Index, and ζ-Potential of HSA-Polymer Aggregates in PBS Determined with DLS (Concentration of HSA = 1 mg/mL, *T* = 37 °C)

polymer/aggregate	*d* [nm] (by number)	DI	ζ-potential (mV)
HSA	6.93 ± 0.28	0.35 ± 0.06	–10.1 ± 1.0
PSSNa_141_ + HSA	9.58 ± 0.26	0.34 ± 0.04	–10.1 ± 2.4
PEG_220_-*b*-PSSNa_54_ + HSA	6.93 ± 0.50	0.22 ± 0.03	–10.4 ± 0.8
PEG_220_-*b*-PSSNa_81_ + HSA	7.64 ± 0.18	0.21 ± 0.06	–13.2 ± 1.7
PEG_220_-*b*-PSSNa_94_ + HSA	9.44 ± 0.51	0.42 ± 0.04	–15.5 ± 1.5
PEG_44_-*b*-PSSNa_79_ + HSA	10.58 ± 1.09	0.33 ± 0.06	–18.1 ± 2.1
PEG_44_-*b*-PSSNa_120_ + HSA	8.85 ± 0.83	0.28 ± 0.06	–16.7 ± 4.1
PEG_44_-*b*-PSSNa_157_ + HSA	9.09 ± 0.56	0.13 ± 0.02	–16.2 ± 1.7

**Table 4:** Thermodynamic Parameters of the Interaction between PEG-*b*-PSSNa Copolymers and Human Serum Albumin (HSA)^a^^b^^c^^d^

	stoichiometry	*K*_a_ (×10^6^ M^–1^)	Δ*H*_a_ (kJ/mol)	Δ*S*_a_ (J/mol/K)	Δ*G*_a_ (kJ/mol)	*K*_d_ (μM)
PSSNa_141_	3.45 ± 0.04	0.769 ± 0.074	47.4 ± 0.8	272 ± 2	–33.6 ± 0.2	1.3 ± 0.12
PEG_220_-*b*-PSSNa_94_	2.45 ± 0.03	0.739 ± 0.059	55.6 ± 0.9	299 ± 3	–33.5 ± 0.2	1.35 ± 0.11
PEG_220_-*b*-PSSNa_81_	1.86 ± 0.03	0.896 ± 0.106	53.1 ± 1.6	292 ± 5	–34 ± 0.3	1.12 ± 0.13
PEG_220_-*b*-PSSNa_54_	1.61 ± 0.03	0.876 ± 0.112	52.3 ± 1.9	289 ± 6	–33.9 ± 0.3	1.14 ± 0.15
PEG_44_-*b*-PSSNa_157_	3.36 ± 0.03	0.642 ± 0.048	54.3 ± 1	293 ± 3	–33.2 ± 0.2	1.56 ± 0.12
PEG_44_-*b*-PSSNa_120_	2.69 ± 0.04	0.539 ± 0.051	58.6 ± 1.8	306 ± 5	–32.7 ± 0.2	1.85 ± 0.17
PEG_44_-*b*-PSSNa_79_	1.75 ± 0.03	0.818 ± 0.093	53.2 ± 1.7	292 ± 5	–33.8 ± 0.3	1.22 ± 0.14

a *K*_a_: affinity constant.

b Δ*H*: enthalpy change.

c Δ*G*: free enthalpy change.

d Δ*S*: entropy change.

**Figure 4:**
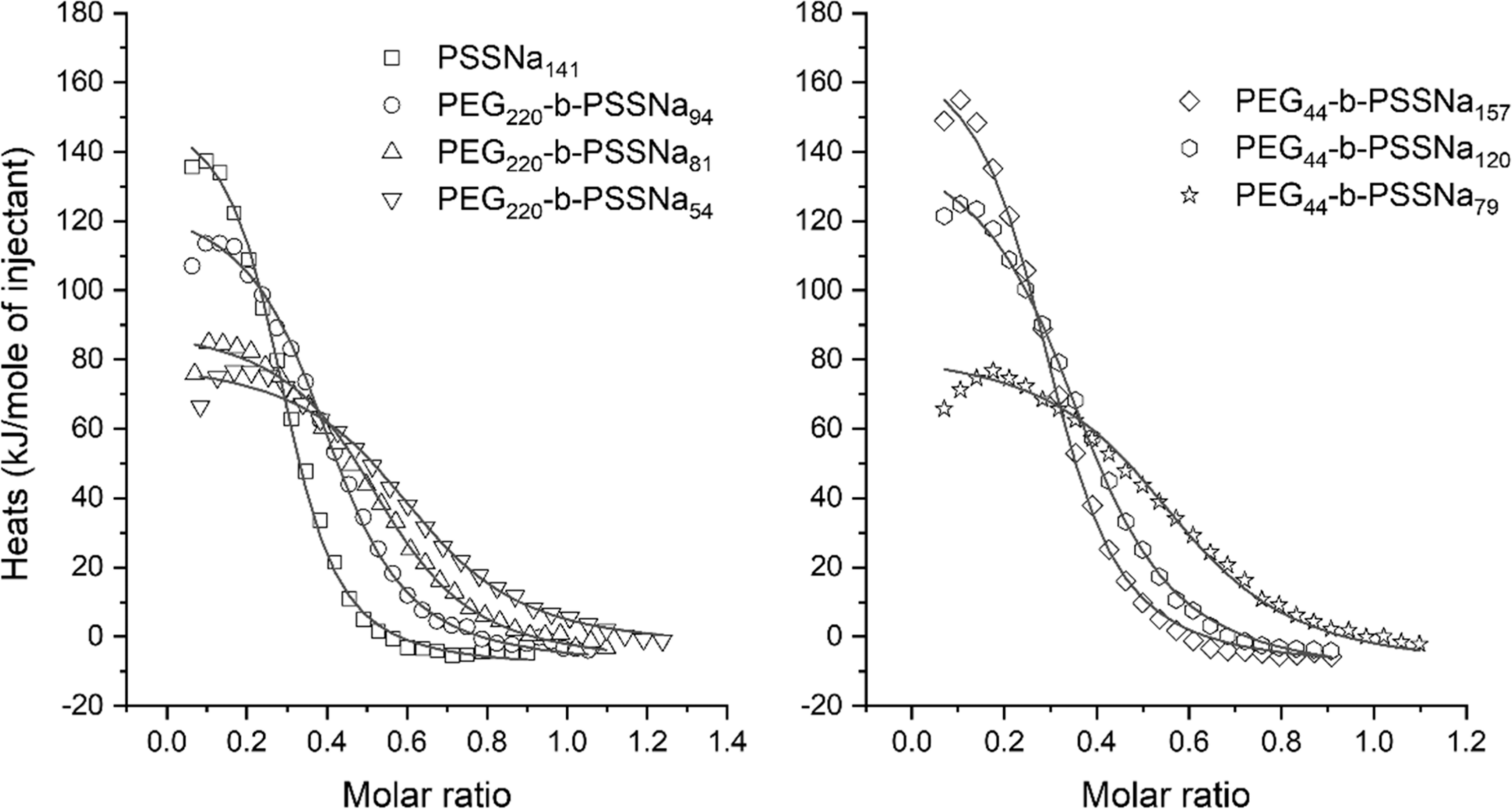
Representative calorimetric isotherms of the binding of PEG-*b*-PSSNa copolymers to HSA. Experiments were carried out in PBS at 37 °C. The lines represent the best fit of the one class of binding sites model to the experimental data.

Thermodynamics of HSA–polymer interactions were studied with ITC (Figures 4 and S4). The process could be analyzed according to the model of one class of binding sites, with the assumption that the ligand was a protein. The HSA/PSSNa stoichiometry is controlled by the length of the PSSNa segment of the macromolecule: it is about 3 for the longer ones, while it is close to 2 for shorter ones (Figure S5). The thermodynamic parameters do not differ significantly for various polymers studied. The process is spontaneous with Δ*G*_a_ about −33 kJ/mol and endothermic. The large entropy increase reflects the release of ions and water molecules on polymer–protein interaction.

## 4 Discussion

Zika virus is an emerging flavivirus responsible for the neurodevelopmental congenital Zika syndrome and has been linked to the neuroinflammatory Guillain–Barré syndrome in adults. Although the number of ZIKV infections has drastically decreased from its peak during the Latin American outbreak of 2015–2017, the threat of a virus resurgence due to genetic drift, human travel, and vector habitat change is real. Moreover, recent experiments conducted in the model that mimics the natural transmission cycle confirm that mutations acquired by the virus may increase transmissibility and pathogenicity.^25^

Currently, there are no drugs or preventive measures available, and therefore there is an urgent need for novel strategies. However, as the virus causes systemic disease after the infection, it is important to consider the systemic administration route. In such a case, a number of issues must be addressed, starting from the interaction between the drug molecule and proteins in the plasma. Here, we studied the interaction of the PEG-*b*-PSSNa with HSA, the most abundant protein in human blood plasma, using DLS and ITC techniques and observed that PEG*-b*-PSSNa polymers form small nanometric, negatively charged complexes. The process is controlled mainly by the electrostatic interactions between the ionic block of polymers modified, to some extent, by the PEG block. These findings corroborate the earlier studies on polyelectrolyte interactions with protein, including HSA.^26−31^ Considering the fact that the isoelectric point of HSA, pI = 5.0, its molecule is negatively charged at physiological pH, which should preclude the occurrence of the attractive electrostatic forces between that protein and PSSNa polyanion. However, it was demonstrated that protein molecules are involved in these interactions due to the presence of positively charged domains on their surfaces.^30^ The protein molecules serve as multivalent counterions for polyanion.^27^ Thus, complexation is associated with releasing small counterions, originally condensed on the polyelectrolyte macromolecule. As shown by ITC experiments, statistically, more than three molecules of HSA are associated with the PSSNa_141_ macromolecule, while statistically less than 2 with PEG_220_-*b*-PSS_54_. The ratio of PSSNa length and stoichiometric ratio is similar for all tested polymers; the mean value is 42. This result can be interpreted as the size of the PSSNa binding site for one molecule of HSA. The significant entropy change confirms the release of a large number of small counterions on complexation. Interestingly, we have observed that the presence of PEG blocks in PEG-*b*-PSSNa polymers (pegylation of PSSNa) affected polymer–protein interactions only slightly. It is known that PEG serves as a highly solvated antifouling material and can act as a protein repellent while coated on surfaces. However, there is very limited knowledge on PEG–protein interaction in aqueous media.^27^ Studies on PEG interaction with bovine serum albumin (BSA) and lysozyme (LYZ) in phosphate-buffered saline indicated that the molecular weight of PEG is crucial in that phenomenon.^27,31^ PEG with high molecular weight can change the microenvironment and conformation of the protein, affecting its activity.^31^ That observation was confirmed in studies on pegylated LYZ. Generally, pegylation increased the protein solubility by more than 11-fold, but high-molecular-weight PEG had a negative effect on protein activity. In our studies, all PEG-*b*-PSSNa polymers were active in the complexation of HSA. Importantly, the PEG-*b*-PSSNa-HSA complexes are negatively charged, like HSA molecules, and their sizes are smaller than 10 nm (with one exception). One can assume that these complexes will be dispersed in aqueous media, possibly also in the blood plasma, and stabilized *via* repulsive electrostatic forces.

## Supporting Information Available

FTIR spectra of PEG_220_-CTA, PSSNa polymer, and PEG_220_-*b*-PSSNa_75_ copolymer (Figure S1); ^1^H NMR spectra of PEG-*b*-PSSNa block copolymers in D_2_O (Figure S2); ATR-FTIR spectra of PEG-*b*-PSSNa block copolymers (Figure S3); dependence of the HSA:PSSNa stoichiometry on the length of PSSNa block of PEG-b-PSSNa macromolecule (Figure S4); ITC measurements for PEG-block-PSSNa copolymers binding to HSA (Figure S5); results of DLS measurements for PEG-*b*-PSSNa block copolymers at various pH values (Table S1); results of DLS measurements for the HSA–polymer aggregates in PBS (Table S2); results of DLS measurements for the BSA-polymer aggregates in PBS (Table S3) (PDF)

## References

[ref1] Lindenbach B. D.; Thiel H.-J.; Rice C. Flaviviridae: The Viruses and Their Replication. Fields Virol. 2007, 1102–1153.

[ref2] Marchette N. J.; Garcia R.; Rudnick A. Isolation of Zika Virus from Aedes Aegypti Mosquitoes in Malaysia. Am. J. Trop. Med. Hyg. 1969, 18, 411–415. 10.4269/ajtmh.1969.18.411. 4976739

[ref3] Dick G. W. A.; Kitchen S.; Haddow A. Zika Virus (I). Isolations and Serological Specificity. Trans. R. Soc. Trop. Med. Hyg. 1952, 46, 509–520. 10.1016/0035-9203(52)90042-4. 12995440

[ref4] Duffy M. R.; Chen T. H.; Hancock W. T.; Powers A. M.; Kool J. L.; Lanciotti R. S.; Pretrick M.; Marfel M.; Holzbauer S.; Dubray C.; Guillaumot L.; Griggs A.; Bel M.; Lambert A. J.; Laven J.; Kosoy O.; Panella A.; Biggerstaff B. J.; Fischer M.; Hayes E. B. Zika Virus Outbreak on Yap Island, Federated States of Micronesia. N. Engl. J. Med. 2009, 360, 2536–2543. 10.1056/NEJMoa0805715. 19516034

[ref5] Musso D.; Bossin H.; Mallet H. P.; Besnard M.; Broult J.; Baudouin L.; Levi J. E.; Sabino E. C.; Ghawche F.; Lanteri M. C.; Baud D. Zika Virus in French Polynesia 2013–14: Anatomy of a Completed Outbreak. Lancet Infect. Dis. 2018, 18, e172–e182. 10.1016/S1473-3099(17)30446-2. 29150310

[ref6] Brasil P.; Pereira J. P.; Moreira M. E.; Nogueira R. M. R.; Damasceno L.; Wakimoto M.; Rabello R. S.; Valderramos S. G.; Halai U. A.; Salles T. S.; Zin A. A.; Horovitz D.; Daltro P.; Boechat M.; Gabaglia C. R.; De Sequeira P. C.; Pilotto J. H.; Medialdea-Carrera R.; Da Cunha D. C.; De Carvalho L. M. A.; Pone M.; Siqueira A. M.; Calvet G. A.; Baiao A. E. R.; Neves E. S.; De Carvalho P. R. N.; Hasue R. H.; Marschik P. B.; Einspieler C.; Janzen C.; Cherry J. D.; De Filippis A. M. B.; Nielsen-Saines K. Zika Virus Infection in Pregnant Women in Rio de Janeiro. N. Engl. J. Med. 2016, 375, 2321–2334. 10.1056/NEJMoa1602412. 26943629 PMC5323261

[ref7] Osorio-De-Castro C. G. S.; Miranda E. S.; De Freitas C. M.; De Camargo K. R.; Cranmer H. H. The Zika Virus Outbreak in Brazil: Knowledge Gaps and Challenges for Risk Reduction. Am. J. Public Health 2017, 107, 960–965. 10.2105/AJPH.2017.303705. 28426311 PMC5425851

[ref8] Haby M. M.; Pinart M.; Elias V.; Reveiz L. Prevalence of Asymptomatic Zika Virus Infection: A Systematic Review. Bull. W. H. O. 2018, 96, 402–413D. 10.2471/BLT.17.201541. 29904223 PMC5996208

[ref9] Adachi K.; Nielsen-Saines K. Zika Clinical Updates: Implications for Pediatrics. Curr. Opin. Pediatr. 2018, 30, 105–116. 10.1097/MOP.0000000000000582. 29176498 PMC5798463

[ref10] Wheeler A. C. Development of Infants with Congenital Zika Syndrome: What Do We Know and What Can We Expect?. Pediatrics 2018, 141, S154–S160. 10.1542/peds.2017-2038D. 29437048 PMC5795516

[ref11] Parra B.; Lizarazo J.; Jiménez-Arango J. A.; Zea-Vera A. F.; González-Manrique G.; Vargas J.; Angarita J. A.; Zuñiga G.; Lopez-Gonzalez R.; Beltran C. L.; Rizcala K. H.; Morales M. T.; Pacheco O.; Ospina M. L.; Kumar A.; Cornblath D. R.; Muñoz L. S.; Osorio L.; Barreras P.; Pardo C. A. Guillain-Barré Syndrome Associated with Zika Virus Infection in Colombia. N. Engl. J. Med. 2016, 375, 1513–1523. 10.1056/NEJMoa1605564. 27705091

[ref12] Bianculli R. H.; Mase J. D.; Schulz M. D. Antiviral Polymers: Past Approaches and Future Possibilities. Macromolecules 2020, 53, 9158–9186. 10.1021/acs.macromol.0c01273.

[ref13] Pirrone V.; Wigdahl B.; Krebs F. C. The Rise and Fall of Polyanionic Inhibitors of the Human Immunodeficiency Virus Type 1. Antiviral Res. 2011, 90, 168–182. 10.1016/j.antiviral.2011.03.176. 21439325

[ref14] Pachota M.; Klysik K.; Synowiec A.; Ciejka J.; Szczubiałka K.; Pyrć K.; Nowakowska M. Inhibition of Herpes Simplex Viruses by Cationic Dextran Derivatives. J. Med. Chem. 2017, 60, 8620–8630. 10.1021/acs.jmedchem.7b01189. 28956914

[ref15] Connor E. F.; Lees I.; Maclean D. Polymers as Drugs—Advances in Therapeutic Applications of Polymer Binding Agents. J. Polym. Sci., Part A: Polym. Chem. 2017, 55, 3146–3157. 10.1002/pola.28703.

[ref16] Milewska A.; Chi Y.; Szczepanski A.; Barreto-Duran E.; Dabrowska A.; Botwina P.; Obloza M.; Liu K.; Liu D.; Guo X.; Ge Y.; Li J.; Cui L.; Ochman M.; Urlik M.; Rodziewicz-Motowidlo S.; Zhu F.; Szczubialka K.; Nowakowska M.; Pyrc K. HTCC as a Polymeric Inhibitor of SARS-CoV-2 and MERS-CoV. J. Virol. 2020, 95, e01622-20 10.1128/jvi.01622-20. PMC7851557 33219167

[ref17] Pyrć K.; Milewska A.; Duran E. B.; Botwina P.; Lopes R.; Arenas-Pinto A.; Badr M.; Mellor R.; Kalber T. L.; Fernandes-Reyes D.; Schätzlein A. G.; Uchegbu I. F. SARS-CoV-2 Inhibition in Human Airway Epithelial Cells Using a Mucoadhesive, Amphiphilic Chitosan That May Serve as an Anti-Viral Nasal Spray bioRxiv 2020, 10.1101/2020.12.10.413609.

[ref18] Soria-Martinez L.; Bauer S.; Giesler M.; Schelhaas S.; Materlik J.; Janus K.; Pierzyna P.; Becker M.; Snyder N. L.; Hartmann L.; Schelhaas M. Prophylactic Antiviral Activity of Sulfated Glycomimetic Oligomers and Polymers. J. Am. Chem. Soc. 2020, 142, 5252–5265. 10.1021/jacs.9b13484. 32105452

[ref19] Zelikin A. N.; Stellacci F. Broad-Spectrum Antiviral Agents Based on Multivalent Inhibitors of Viral Infectivity. Adv. Healthcare Mater. 2021, 10, 2001433 10.1002/adhm.202001433. PMC7995163 33491915

[ref20] Botwina P.; Obłoza M.; Szczepanski A.; Szczubiałka K.; Nowakowska M.; Pyrć K. In Vitro Inhibition of Zika Virus Replication with Poly(Sodium 4-Styrenesulfonate). Viruses 2020, 12, 926 10.3390/v12090926. 32842540 PMC7551931

[ref21] Veronese F. M.; Pasut G. PEGylation, Successful Approach to Drug Delivery. Drug Discovery Today 2005, 10, 1451–1458. 10.1016/s1359-6446(05)03575-0. 16243265

[ref22] Yadav D.; Dewangan H. K. PEGYLATION: An Important Approach for Novel Drug Delivery System. J. Biomater. Sci., Polym. Ed. 2021, 32, 266–280. 10.1080/09205063.2020.1825304. 32942961

[ref23] Reed L. J.; Muench H. Simple Method of Estimating Fifty per Cent Endpoints. Am. J. Epidemiol. 1938, 27, 493–497. 10.1093/oxfordjournals.aje.a118408.

[ref24] Synowiec A.; Gryniuk I.; Pachota M.; Strzelec Ł.; Roman O.; Kłysik-Trzciańska K.; Zając M.; Drebot I.; Gula K.; Andruchowicz A.; Nowakowska M.; Pyrc K.; et al. Cat Flu: Broad Spectrum Polymeric Antivirals. Antiviral Res. 2019, 170, 104563 10.1016/j.antiviral.2019.104563. 31325462

[ref25] Regla-Nava J. A.; Wang Y. T.; Fontes-Garfias C. R.; Liu Y.; Syed T.; Susantono M.; Gonzalez A.; Viramontes K. M.; Verma S. K.; Kim K.; Landeras-Bueno S.; Huang C. T.; Prigozhin D. M.; Gleeson J. G.; Terskikh A. V.; Shi P. Y.; Shresta S. A Zika Virus Mutation Enhances Transmission Potential and Confers Escape from Protective Dengue Virus Immunity. Cell Rep. 2022, 39, 110655 10.1016/j.celrep.2022.110655. 35417697 PMC9093040

[ref26] Da Silva F. L. B.; Jönsson B. Polyelectrolyte-Protein Complexation Driven by Charge Regulation. Soft Matter 2009, 5, 2862–2868. 10.1039/b902039j.

[ref27] Wu J.; Zhao C.; Lin W.; Hu R.; Wang Q.; Chen H.; Li L.; Chen S.; Zheng J. Binding Characteristics between Polyethylene Glycol (PEG) and Proteins in Aqueous Solution. J. Mater. Chem. B 2014, 2, 2983–2992. 10.1039/c4tb00253a. 32261674

[ref28] Yu S.; Xu X.; Yigit C.; Van Der Giet M.; Zidek W.; Jankowski J.; Dzubiella J.; Ballauff M. Interaction of Human Serum Albumin with Short Polyelectrolytes: A Study by Calorimetry and Computer Simulations. Soft Matter 2015, 11, 4630–4639. 10.1039/c5sm00687b. 25959568

[ref29] Minsky B. B.; Dubin P. L.; Kaltashov I. A. Electrostatic Forces as Dominant Interactions Between Proteins and Polyanions: An ESI MS Study of Fibroblast Growth Factor Binding to Heparin Oligomers. J. Am. Soc. Mass Spectrom. 2017, 28, 758–767. 10.1007/s13361-017-1596-0. 28211013 PMC5808462

[ref30] Wiig H.; Kolmannskog O.; Tenstad O.; Bert J. L. Effect of Charge on Interstitial Distribution of Albumin in Rat Dermis in Vitro. J. Physiol. 2003, 550, 505–514. 10.1113/jphysiol.2003.042713. 12766239 PMC2343033

[ref31] Morgenstern J.; Baumann P.; Brunner C.; Hubbuch J. Effect of PEG Molecular Weight and PEGylation Degree on the Physical Stability of PEGylated Lysozyme. Int. J. Pharm. 2017, 519, 408–417. 10.1016/j.ijpharm.2017.01.040. 28130198

