# Supplementary material for: Poly(ethylene glycol)-block-poly(sodium 4-styrenesulfonate) Copolymers as Efficient Zika Virus Inhibitors: In Vitro Studies
Source: ACS Omega. 2023 Feb 9;8(7):6875–83. doi: 10.1021/acsomega.2c07610 (PMC9948194; doi:10.1021/acsomega.2c07610)
Supplement: Supplementary file 1 — ao2c07610_si_001.pdf [file ao2c07610_si_001.pdf]

Supporting Information

Poly(ethylene glycol) – *block-* Poly(Sodium 4-Styrene sulfonate)) Copolymers as Efficient Zika Virus Inhibitors – *In vitro* Studies

Paweł Botwina [1,2], Magdalena Obłoza[3], Piotr Bonarek[4], Krzysztof Szczubiałka[3], Krzysztof Pyrć[1,*] and Maria Nowakowska[3,*]

[1] Virogenetics Laboratory of Virology, Malopolska Centre of Biotechnology, Jagiellonian University, Krakow, Poland
[2] Microbiology Department, Faculty of Biochemistry, Biophysics and Biotechnology, Jagiellonian University, Krakow, Poland
[3] Department of Physical Chemistry, Faculty of Chemistry, Jagiellonian University, Krakow, Poland
[4] Department of Physical Biochemistry, Faculty of Biochemistry, Biophysics and Biotechnology, Jagiellonian University, Krakow, Poland

***Correspondence:** k.a.pyrc@uj.edu.pl **(K.P.);** nowakows@chemia.uj.edu.pl **(M.N.)**

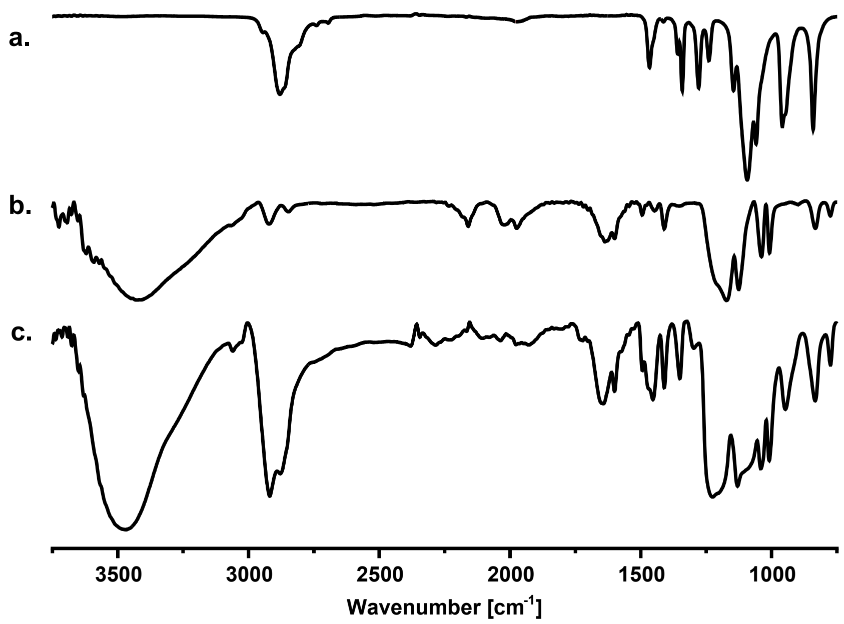

Figure S1. FT-IR spectra of PEG_{220}-CTA (a), PSSNa polymer (b), and PEG_{220}-*b*-$PSSNa_{81}$copolymer (c).

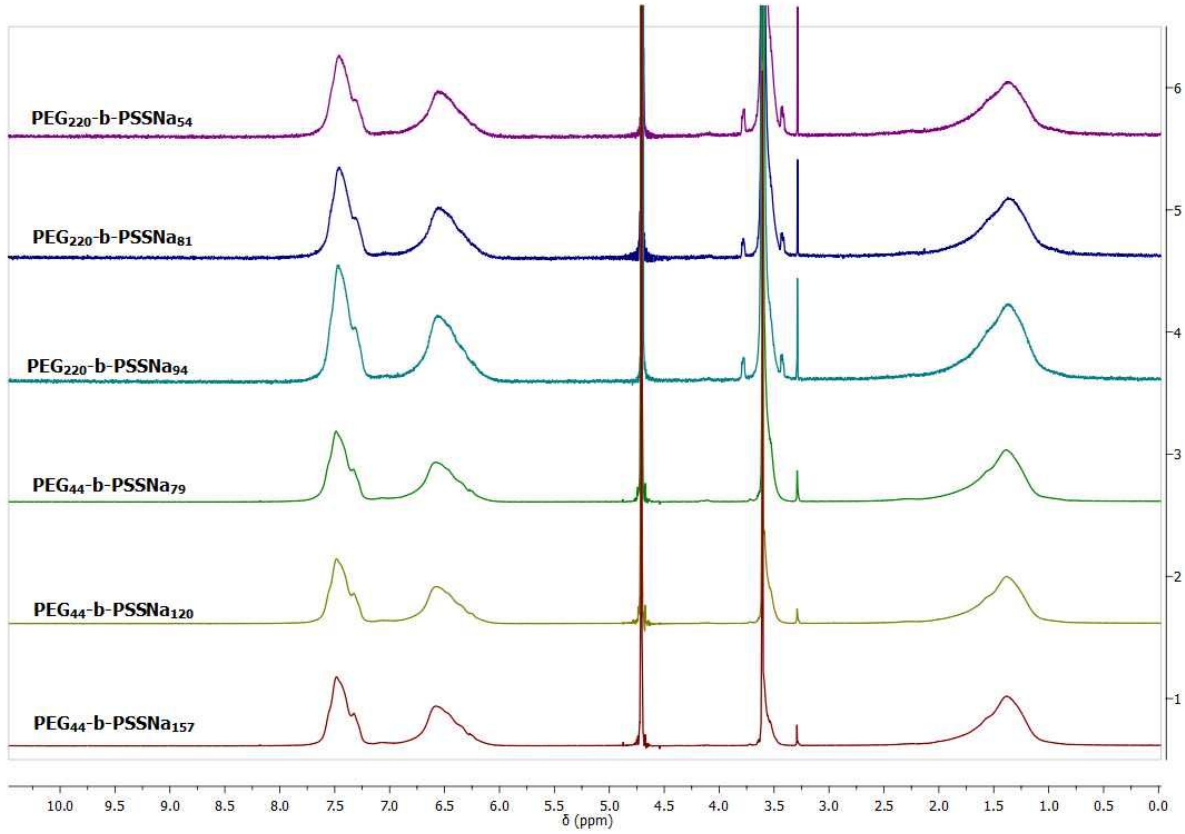

Figure S2. ^{1}H NMR spectra of PEG-*b*-PSSNa copolymers in D_2O.

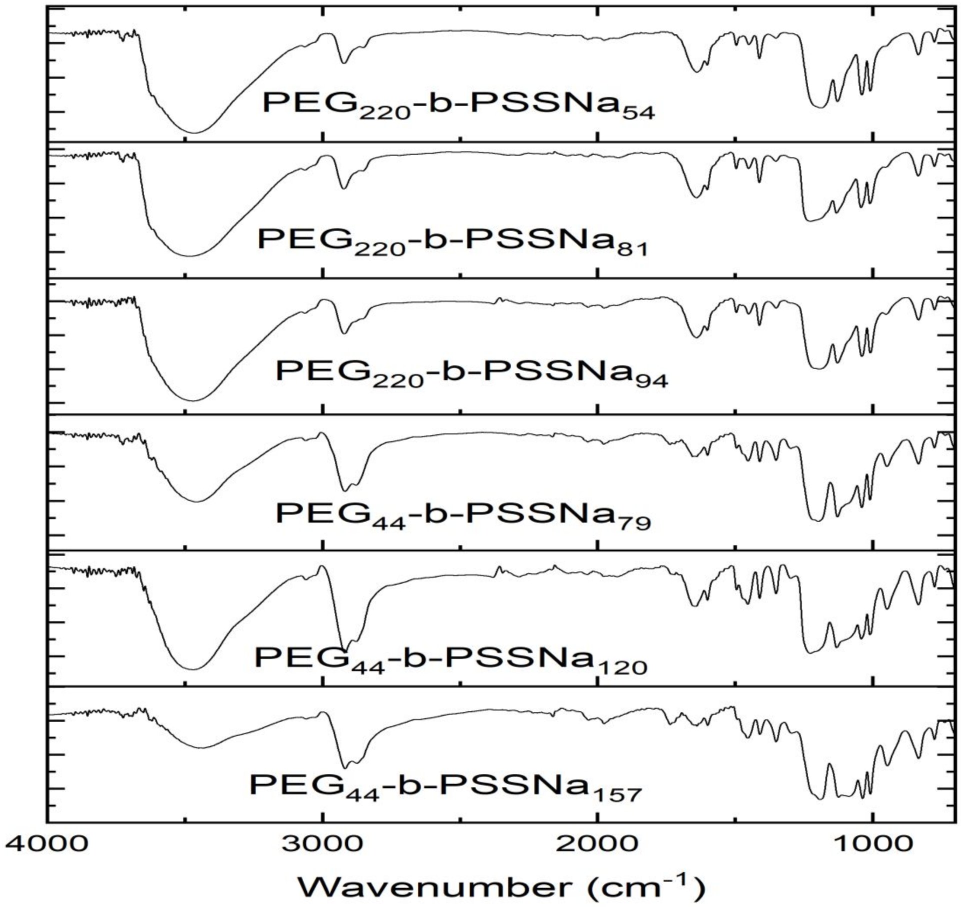

Figure S3. ATR-FT-IR spectra of PEG-*b*- PSSNa block copolymers.

Table S1. Dimension, dispersity index and zeta potential of polymers in different pH-media (diluted solution of HCl/NaOH) determined with DLS (concentration of polymer/copolymer = 1 mg/mL, T= 37°C).

pH	**PEG_{44}-b-$PSSNa_{157}$**			**PEG_{220}-b-$PSSNa_{94}$**		
	d [nm] (by number)	Đ	Zeta potential (mV)	d [nm] (by number)	Đ	Zeta potential (mV)
1	6.85 ± 0.08	0.41±0.13	-22,7 ± 5.2	6.74 ± 0.25	0.34±0.08	-12.7 ± 5.2
2	6.86 ± 0.90	0.43±0.17	-24.8 ± 4.1	5.42 ± 0.49	0.80±0.20	-21.1 ± 0.44
3	4.50 ± 0.11	0.72±0.24	-40.0 ± 10.5	5.27 ± 0.69	0.43±0.15	-35.3 ± 2.6
4	1.49 ± 0.39	1.00±0.00	-45.9 ± 2.1	1.11 ± 0.42	0.93±0.11	-37.9 ± 6.1
5	1.49 ± 0.59	1.00±0.00	-22.4 ± 7.0	1.19 ± 0.17	0.89±0.16	-41.8 ± 5.0
6	0.97 ± 0.22	1.00±0.00	-36.2 ± 7.2	3.49 ± 0.01	0.84±0.23	-37.9 ± 1.9
7	0.82 ± 0.16	0.82±0.24	-35.7 ± 3.9	4.15 ± 1.44	1.00±0.00	-47.2 ± 1.6
8	0.97 ± 0.32	1.00±0.00	-46.4 ± 5.8	2.55 ± 1.62	0.99±0.01	-34.9 ± 10.3
9	1.04 ± 0.10	0.98±0.04	-31.9 ± 1.7	1.06 ± 0.59	0.94±0.11	-31.2 ± 5.7
10	1.01 ± 0.25	0.29±0.09	-45.8 ± 5.1	3.26 ± 1.22	0.63±0.15	-35.5 ± 0.99
11	2.48 ± 0.96	0.83±0.30	-40.9 ± 2.8	3.72 ± 0.32	0.73±0.35	-33.1 ± 6.2
12	7.89 ± 0.51	0.23±0.08	-28.7 ± 16.7	6.14 ± 0.48	0.47±0.25	-27.4 ± 3.0
13	7.64 ± 0.70	0.60±0.21	-19.4 ± 2.0	4.94 ± 1.13	0.97±0.04	-13.5 ± 3.3
14	7.35 ± 0.60	0.54±0.26	-20.3 ± 2.1	5.58 ± 0.13	0.96±0.14	-15.8 ± 3.6

Table S2. Dimension, dispersity index and zeta potential of HSA-polymer aggregates in PBS determined with DLS (concentration of HSA = 1 mg/mL, T= 37°C) after 24 h.

Polymer/aggregate	d [nm] (by number)	Đ	Zeta potential (mV)
**HSA**	5.93 ± 0.29	0.11 ± 0.02	-9.07 ± 1.0
**$PSSNa_{141}$ + HSA**	7.56 ± 0.27	0.21 ± 0.01	-14.5 ± 2.3
**PEG_{220}-b-$PSSNa_{54}$ + HSA**	7.12 ± 0.56	0.19 ± 0.03	-12.3 ± 2.5
**PEG_{220}-b-$PSSNa_{81}$ + HSA**	7.28 ± 0.16	0.17 ± 0.01	-12.1 ± 2.0
**PEG_{220}-b-$PSSNa_{94}$ + HSA**	8.92 ± 0.39	0.14 ± 0.01	-14.1 ± 2.1
**PEG_{44}-b-$PSSNa_{79}$ + HSA**	6.92 ± 0.06	0.15 ± 0.02	-15.7 ± 5.1
**PEG_{44}-b-$PSSNa_{120}$ + HSA**	8.32 ± 0.08	0.18 ± 0.04	-11.4 ± 0.7
**PEG_{44}-b-$PSSNa_{157}$ + HSA**	8.88 ± 0.18	0.11 ± 0.02	-13.5 ± 2.4

Table S3. Size, dispersity index and zeta potential of BSA-polymer aggregates in PBS determined with DLS (concentration of BSA/polymer = 1 mg/mL, T = 37°C).

Polymer/aggregate	d [nm] (by number)	Đ	Zeta potential (mV)
**BSA**	5.90 ± 0.28	0.21 ± 0.09	-9.1 ± 1.2
**$PSSNa_{141}$ + BSA**	8.06 ± 0.52	0.22 ± 0.03	-15.5 ± 2.4
**PEG_{220}-b-$PSSNa_{54}$ + BSA**	7.32 ± 0.31	0.20 ± 0.03	-11.6 ± 0.8
**PEG_{220}-b-$PSSNa_{81}$ + BSA**	7.73 ± 0.51	0.15 ± 0.06	-13.2 ± 1.7
**PEG_{220}-b-$PSSNa_{94}$ + BSA**	8.72 ± 0.45	0.15 ± 0.02	-15.3 ± 2.1
**PEG_{44}-b-$PSSNa_{79}$ + BSA**	6.95 ± 0.45	0.33 ± 0.06	-16.4 ± 3.5
**PEG_{44}-b-$PSSNa_{120}$ + BSA**	8.22 ± 0.66	0.28 ± 0.06	-14.9 ± 2.6
**PEG_{44}-b-$PSSNa_{157}$ + BSA**	9.46 ± 0.55	0.14 ± 0.04	-17.5 ± 2.5

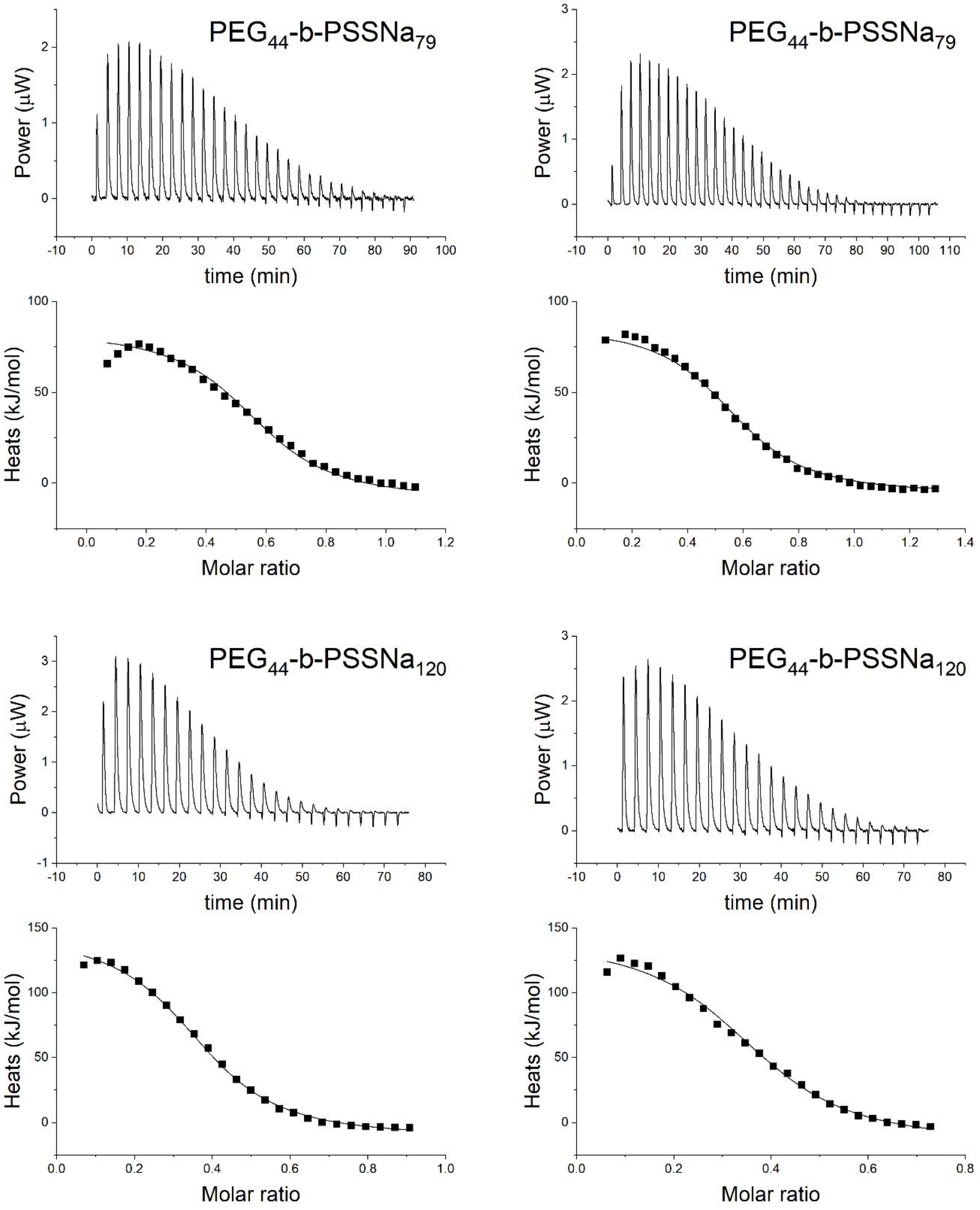
PEG44-b-PSSNa79
PEG44-b-PSSNa79
Power (μW)
time (min)
Heats (kJ/mol)
Molar ratio
PEG44-b-PSSNa120
PEG44-b-PSSNa120
Power (μW)
time (min)
Heats (kJ/mol)
Molar ratio

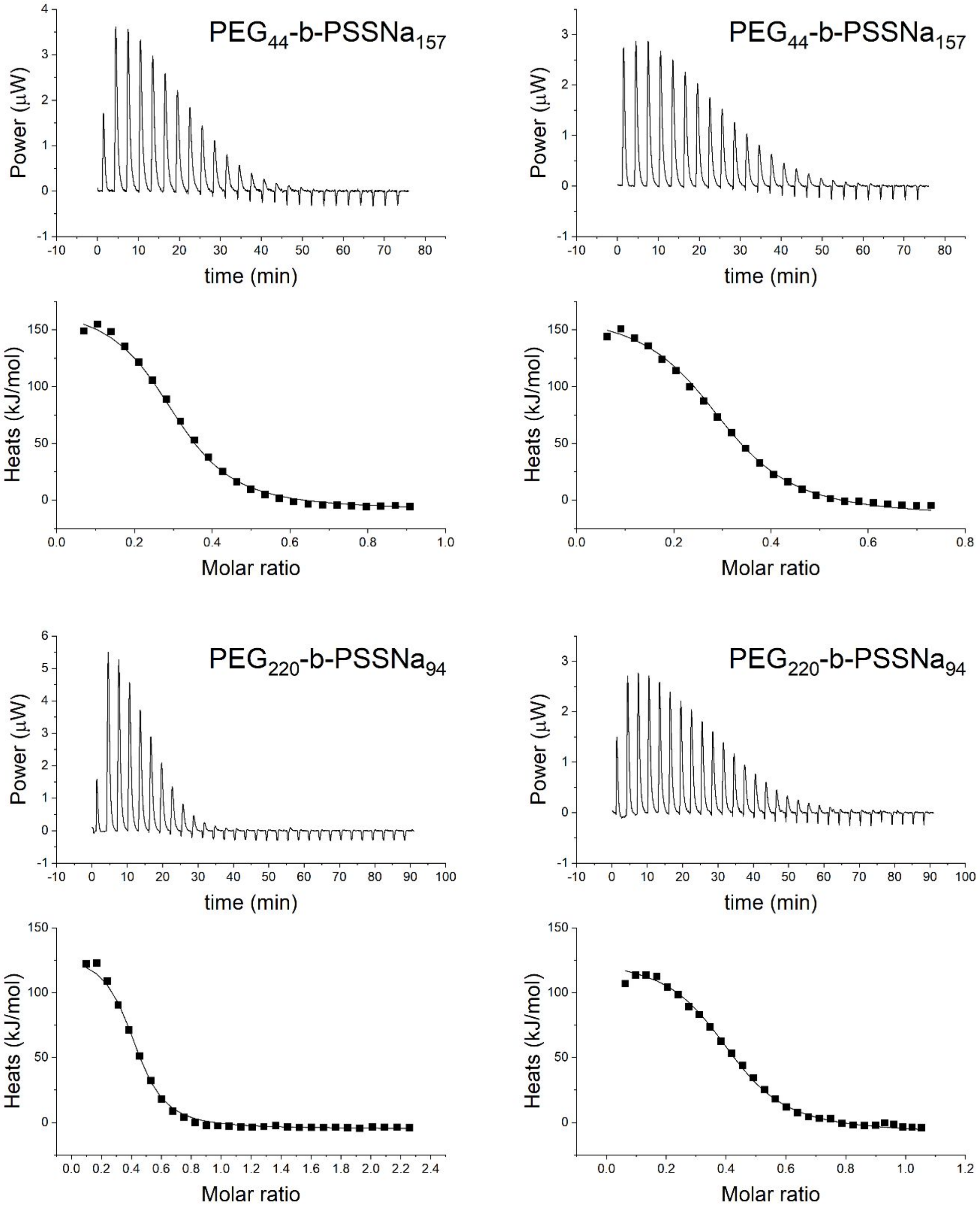
PEG44-b-PSSNa157
Power (µW)
time (min)
Heats (kJ/mol)
Molar ratio
PEG44-b-PSSNa157
Power (µW)
time (min)
Heats (kJ/mol)
Molar ratio
PEG220-b-PSSNa94
Power (µW)
time (min)
Heats (kJ/mol)
Molar ratio
PEG220-b-PSSNa94
Power (µW)
time (min)
Heats (kJ/mol)
Molar ratio

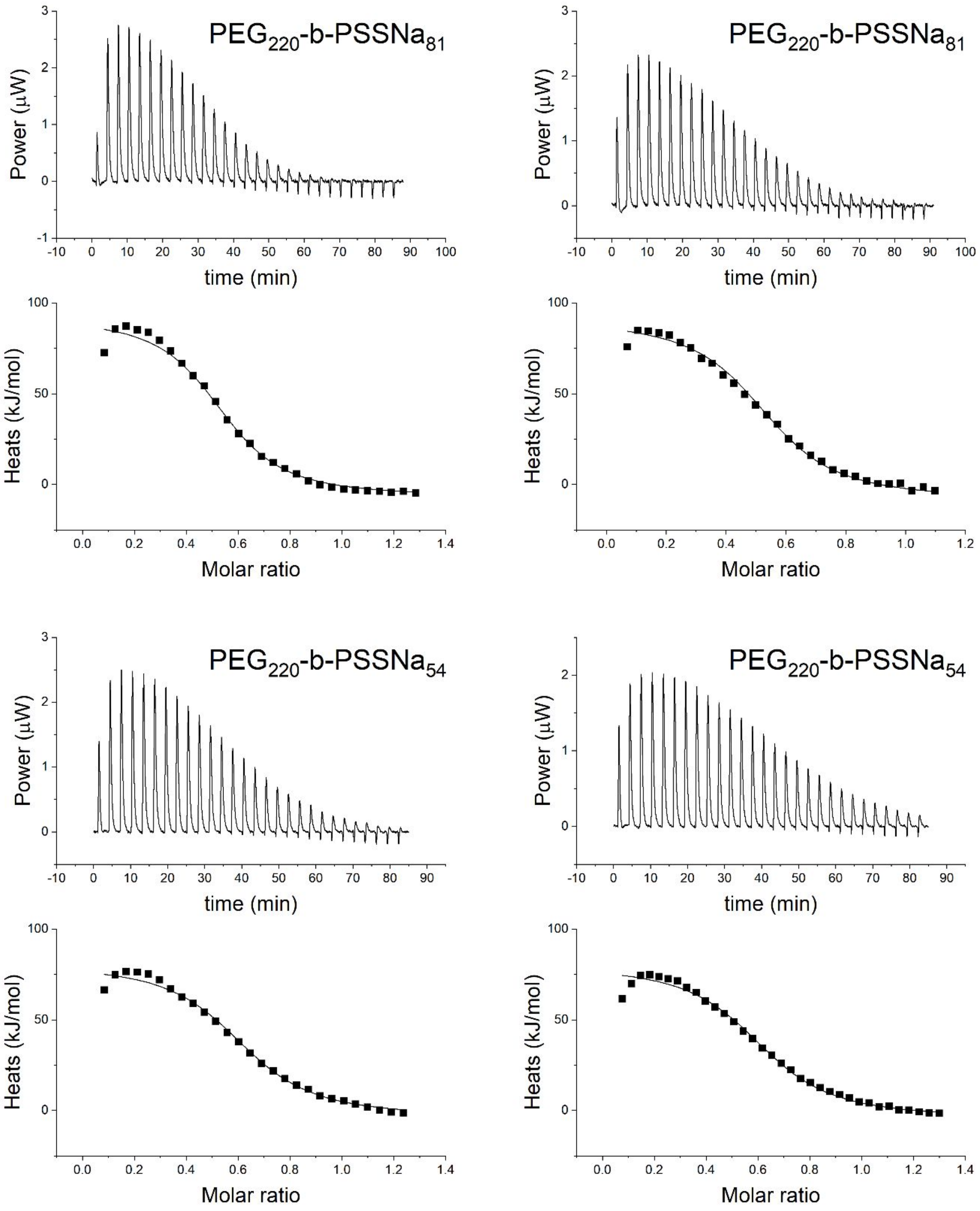
PEG_{220}-b-$PSSNa_{81}$
PEG_{220}-b-$PSSNa_{81}$
PEG_{220}-b-$PSSNa_{54}$
PEG_{220}-b-$PSSNa_{54}$
Power (μW)
time (min)
Heats (kJ/mol)
Molar ratio

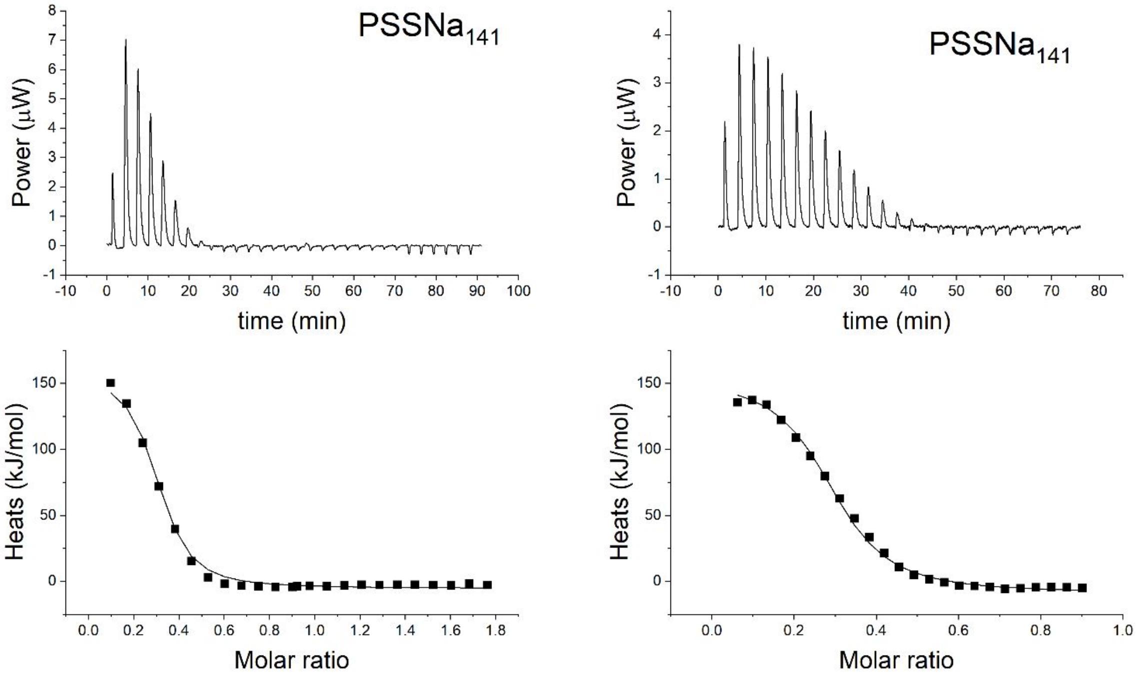

Figure S4. ITC measurements: raw data, calorimetric isotherms of the binding of PEG-block-PSSNa copolymers to HSA. Experiments were carried out in PBS at 37°C. The lines represent the best fit of the one class of binding sites model to the experimental data.

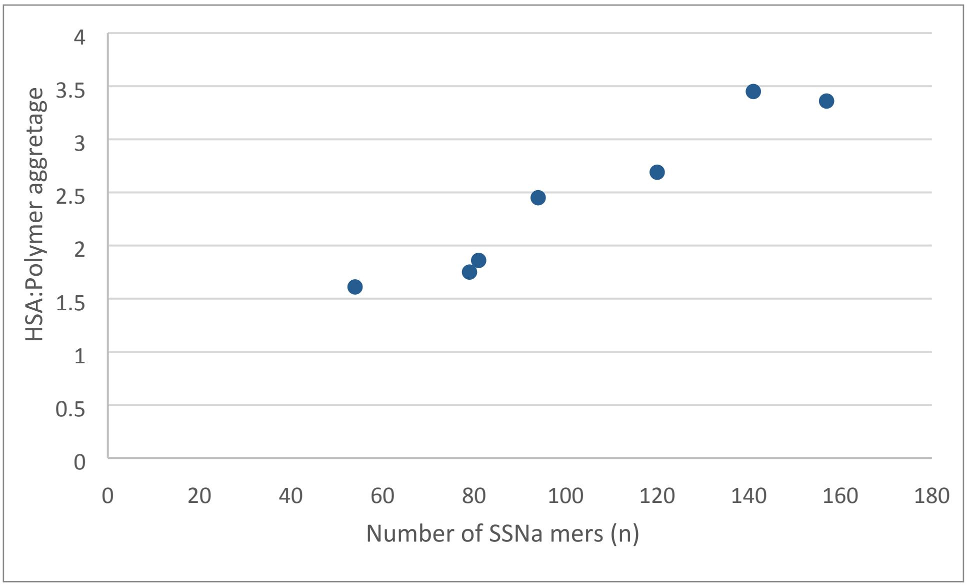

Figure S5. Dependence of HSA:PSSNa stoichiometry on the length of PSSNa block of PEG-*b*-PSSNa macromolecule
